# HCMV-Infected Cells Maintain Efficient Nucleotide Excision Repair of the Viral Genome while Abrogating Repair of the Host Genome

**DOI:** 10.1371/journal.ppat.1003038

**Published:** 2012-11-29

**Authors:** John M. O'Dowd, Anamaria G. Zavala, Celeste J. Brown, Toshio Mori, Elizabeth A. Fortunato

**Affiliations:** 1 Department of Biological Sciences and Center for Reproductive Biology, University of Idaho, Moscow, Idaho, United States of America; 2 Radioisotope Research Center, Nara Medical University School of Medicine, Kashihara, Nara, Japan; University of Wisconsin-Madison, United States of America

## Abstract

Many viruses subvert the host cell's ability to mount and complete various DNA damage responses (DDRs) after infection. HCMV infection of permissive fibroblasts activates host DDRs at the time of viral deposition and during replication, but the DDRs remain uncompleted without arrest or apoptosis. We believe this was in part due to partitioning of the damage response and double strand break repair components. After extraction of soluble proteins, the localization of these components fell into three groups: specifically associated with the viral replication centers (RCs), diffused throughout the nucleoplasm and excluded from the RCs. Others have shown that cells are incapable of processing exogenously introduced damage after infection. We hypothesized that the inability of the cells to process damage might be due to the differential association of repair components within the RCs and, in turn, potentially preferential repair of the viral genome and compromised repair of the host genome. To test this hypothesis we used multiple strategies to examine repair of UV-induced DNA damage in mock and virus-infected fibroblasts. Comet assays indicated that repair was initiated, but was not completed in infected cells. Quantitative analysis of immunofluorescent localization of cyclobutane pyrimidine dimers (CPDs) revealed that after 24 h of repair, CPDs were significantly reduced in viral DNA, but not significantly changed in the infected host DNA. To further quantitate CPD repair, we developed a novel dual-color Southern protocol allowing visualization of host and viral DNA simultaneously. Combining this Southern methodology with a CPD-specific T4 endonuclease V alkaline agarose assay to quantitate repair of adducts, we found efficient repair of CPDs from the viral DNA but not host cellular DNA. Our data confirm that NER functions in HCMV-infected cells and almost exclusively repairs the viral genome to the detriment of the host's genome.

## Author Summary

Human cytomegalovirus (HCMV) is a leading cause of birth defects. This may be due in part to this virus' ability to inflict specific damage to its host's DNA, combined with the disruption of an infected cell's ability to repair damage. Earlier studies found that components of the cell's repair machinery were differentially associated with the HCMV viral replication centers in the nucleus. Experiments here extend this observation to include components of the machinery involved in UV lesion repair. We hypothesized that association of components of the DNA repair machinery within the viral replication centers could favor the repair of viral DNA, but more importantly, be detrimental to the repair of cellular DNA. Infected cells were irradiated and examined for repair by three different methods. In the course of this study, we developed a new technique allowing simultaneous evaluation of both the viral and host genomes in an infected cell. These experiments found rapid, selective removal of UV lesions from the viral and not the cellular DNA within infected cells. Our results indicate the differential association of certain cellular repair proteins with this virus may have far-reaching implications in the disease pathogenesis of HCMV infection.

## Introduction

Human Cytomegalovirus (HCMV) is among the leading causes of birth defects in the United States, affecting an estimated 8000 children per year [1]. Each year ∼1% of all newborns are congenitally infected with HCMV. Of these infants, 5–10% manifest signs of serious neurological defects at birth [2]–[5], with an additional 10–15% subsequently suffering consequences by age five. Recent literature also points to HCMV as a contributing agent for the development of certain types of cancers (for review see [6], [7]). Studies of HCMV infection in non-permissive cells indicate that HCMV can also act as a mutagen [8]–[10], inducing “hit and run” damage.

There is significant evidence that non-specific chromosomal aberrations and damage to the mitotic apparatus can occur in cells infected with a variety of human DNA and RNA viruses (see [11] for review). Yet, only two viruses, the oncogenic adenoviruses (Ad) and HCMV, have been found to cause site-specific chromosomal damage [11]–[13]. We have shown that HCMV is able to induce specific damage in chromosome 1 at two loci, 1q23 and 1q42 [12], [13], as early as 3 h post infection (hpi). In contrast to Ad type 12 [14], [15], induction of specific breaks by HCMV does not require *de novo* viral protein expression. Viral entry into the cell is sufficient to cause the specific breaks. It is also clear from the literature that many viruses interact with their hosts' DNA damage response (DDR) signaling molecules and repair machinery, often triggering responses upon initial entry and deposition of the genome in the nucleus or through successive rounds of replication. Some viruses are reported to utilize this initial DDR response to optimize infection, while others have been found to thwart it (as reviewed in [16], [17]). Work from our lab and others [18]–[20] has shown that host DDRs are activated both at the point of viral deposition and during late phase replication of HCMV in permissive fibroblasts, although the importance of this activation for establishing a fully permissive infection remains unclear. During HCMV infection, DDRs are not finished, resulting in incomplete repair without arrest or apoptosis. We have shown this is due, at least in part, to a differential association of the repair machinery components into the viral replication centers (RCs). After extraction of soluble proteins, we determined three categories of association: specifically associated within RCs, diffused throughout the nucleoplasm and excluded from the RCs [18].

These earlier studies demonstrated specific viral associations with key players in the DDR pathways. Other studies have examined the capability of infected cells (or cells expressing specific viral proteins) to repair different types of damage after infection and found both increases and decreases in the ability to repair induced damage [21]–[40]. However, these earlier studies looked at total cellular DNA and did not examine repair of the viral and host genomes separately within these cells.

We hypothesized that after the RCs were established, association of components of the DNA repair machinery within the RCs of the virus could favor viral repair, but more importantly, be detrimental to repair of the cellular DNA. To the best of our knowledge, our experiments in HCMV-infected cells are the first to examine repair in the host and viral DNA separately and the possibility of preferential repair in the viral DNA. To test our hypothesis, exogenous DNA damage was introduced into cells (UV dimers) via UVC (hereafter UV) irradiation. Analysis of damage repair used comet assays, immunofluorescent localization (IF) of UV-induced cyclopyrimidine dimers (CPDs) and T4 endonuclease V alkaline agarose (T4) assays. These studies found that HCMV-infected cells, although capable of mounting a damage response to UV irradiation, were unable to completely repair all of the exogenously introduced DNA damage. *In situ* localization of the CPDs clearly showed that the residual damage detected in these cells was found entirely within the cellular DNA. Moreover, dual-color T4 assays revealed proficient repair of CPDs from viral DNA but defective repair of host DNA within infected cells. Thus, there was selective repair of DNA damage in viral when compared to cellular DNA in permissively infected fibroblasts, indicating that association of the host's DNA repair machinery with HCMV RCs has detrimental consequences for the host.

## Results

### Tight association with HCMV viral RCs was found for some, but not all, of the nucleotide excision repair (NER) proteins in infected fibroblasts

Our previous work reported tight association of some, but not all, of the ATM-mediated double strand break (DSB) and ATR- mediated stalled replication fork response proteins with HCMV viral RCs within the nucleus of permissively infected cells by 48 hpi [18]. These studies were performed using “extraction first” procedures [41], which differ from the more common “fix first” technique which uses formaldehyde to initially fix proteins in place, followed by permeabilization in detergent to allow access of antibodies (Abs) into the cell. This “fix first” methodology allows for visualization of the entire complement of a given protein within the cell, regardless of how tightly or loosely it is associated with a given compartment. By contrast, an “extraction first” protocol initially treats cells with detergent and then fixes them in formaldehyde [41]. Initial extraction removes proteins that are not attached to the chromatin or scaffolding substructure of the nucleus, providing a clearer view of proteins associated with a given compartment or structure. It is often used in the visualization of DSB repair foci in damaged cells, as only a fraction of the entire protein complement will relocalize to sites of damage.

Performing these two fixation/extraction procedures provided valuable information regarding the nature of protein interactions in infected cells. First, we concluded that the majority of a protein was tightly associated with the RCs if it was distinctly localized within the RCs using only “fix first” protocols. Second, if we saw more distinct localization of a protein with the RCs after “extraction first” conditions we inferred that only a portion of the protein was tightly associated with these centers. It should be noted that the proportion not tightly associated with the RCs was also not tightly associated with the host DNA. Lastly, we concluded the protein was not specifically associated or excluded from these centers when no clear localization to the RCs occurred using either “fix first” or “extraction first” conditions.

**Figure 1:**
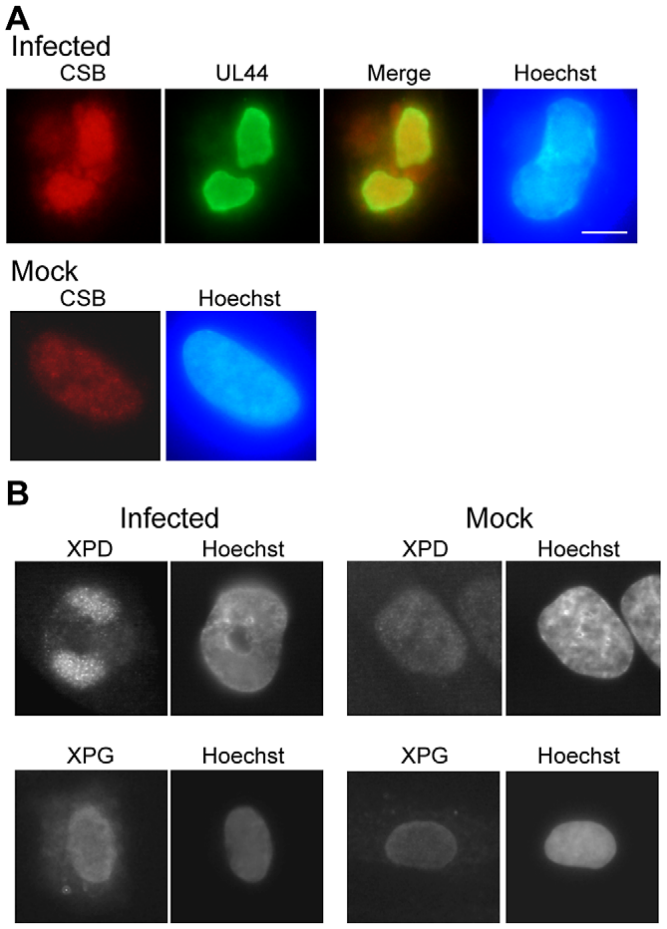
Certain NER proteins were tightly associated with HCMV RCs. A) HFFs were mock- or virus-infected at an MOI of 5 for 48 h and harvested for IF analysis. Coverslips were treated using extract-first conditions (as described in Materials and Methods) and then stained simultaneously for CSB (red) and the viral processivity factor, UL44 (green). Overlay of the two images shows almost complete overlap of the signals (indicating tight association of CSB) in the infected cells. Staining of mock-infected cells shows nuclear localization of CSB. DNA was counterstained with Hoechst (blue). Scalebar on all images = 5 µm. B) Cells were stained for XPD (using “fix first conditions) in the top panels or for XPG (using “extract-first” conditions) in the bottom panels. Mock-infected cells again show nuclear localization of these antigens.

A similar pattern of selective association of nucleotide excision repair (NER) proteins was found in permissively infected HFFs as had been observed for the DSB repair proteins [18]. Figure 1a shows an example of tight association of the Cockaine's Syndrome B (CSB) protein with the viral RCs within the nucleus (as evidenced by colocalization with the viral processivity factor, UL44). Figure 1b shows two other NER proteins, illustrating either tight association with the RCs (XPD) and an example of diffused nuclear staining (XPG). Figure 1 also illustrates the nuclear localization of these three repair proteins in mock-infected cells as a control. In addition, a summary of all the NER-associated proteins tested for localization after infection is given in Table 1. Table 1 indicates the localization of NER proteins using “fix first” or “extraction first” conditions, as indicated (note: it is often difficult to use rabbit primary Abs at 48 hpi using “fix first” conditions due to non-specific binding to the virus-encoded Fc receptor in the cytoplasm).

**Table 1:** Localization of NER proteins within the nucleus.

NER Protein	Localization with respect to viral RCs in the nucleus	
	Fix First Procedure	Extraction First Procedure
XPA	Diffused throughout the nucleus	Diffused throughout the nucleus
XPB*	Associated	Associated
XPC	ND	Associated
XPD*	Associated	Associated
XPF	Diffused throughout the nucleus	Associated
XPG	Diffused throughout the nucleus	Diffused throughout the nucleus
ERCC1	ND	Associated
CSB	ND	Associated

HFFs were infected at an MOI of 5 for 48 h and harvested for IF analysis. Coverslips were treated using fix-first or extract-first conditions (as described in Materials and Methods).

* indicates XPB and XPD showed tight association regardless of fixation conditions. ND = not determined.

The differential localization we observed with the proteins crucial to NER within the RCs led us to hypothesize that the repair of viral DNA could be favored, potentially to the detriment of cellular DNA. This hypothesis was tested using UV irradiation of HCMV infected cells.

### Repair of UV dimers was initiated in HCMV infected cells but was not completed

The first method used to test our hypothesis and visualize repair of UV-induced damage was the single cell gel electrophoresis or comet assay system [42]–[45]. The comet assay has been used historically in the literature to analyze repair of UVC-induced damage. Comet tails are visualized by staining with the fluorescent DNA intercalating agent SYBR Green, which binds to both ss- and dsDNA. Although comet tails could represent other alkali-labile forms of damage, the literature suggests that the very large proportion (>90%) of damage observed in cells irradiated with low doses of UVC irradiation (similar to our studies) are UV dimers ([46]–[48] and references within).

Tails at early timepoints are believed to be the result of initial incision events associated with NER processing of UV dimers. These incisions (strand breaks) allow uncoiling/relaxation of the chromatin to occur. Electrophoretically induced migration of the uncoiled/relaxed DNA is visualized as the formation of a comet tail (as reviewed in [49]). Therefore, only cells capable of initiating repair will have comet tails following UV irradiation [46]. Over a timecourse, successful repair is demonstrated by a decrease in both the number of cells with tails and the % DNA in the tails. Importantly, it has been shown convincingly that cells deficient in NER proteins, e.g. XP proteins, do not form tails above background levels in comet assays after UVC irradiation due to lack of incision events ([46], [47] and references within).

**Figure 2:**
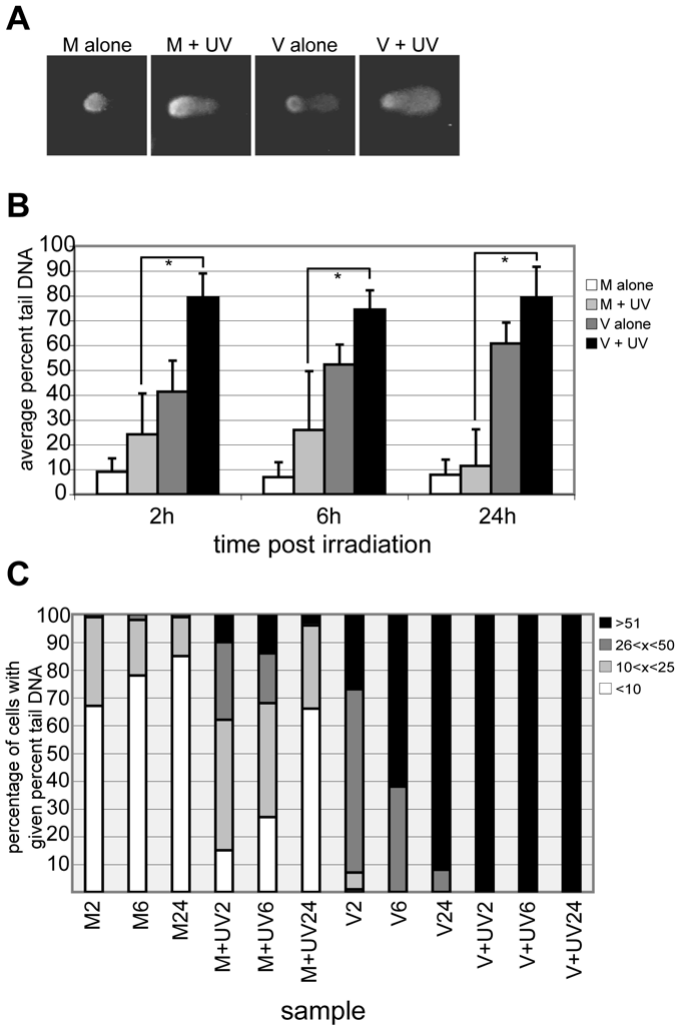
Comet analysis revealed NER repair was initiated in virus-infected cells. HFFs were mock- or virus-infected at an MOI of 5 and irradiated at 48 hpi at a dose of 50 J/m^2^ or left unirradiated. At 2,6 and 24 h p irradiation, cells were harvested for comet analysis. A) Examples from each sample group are shown at 2 hp irradiation. B) Using VisComet, 50–100 cells/sample were analyzed for M alone (white bars), M+UV (light grey bars), V alone (dark grey bars) and V+UV (black bars) samples (except for V+UV 2 h, which had 37 cells analyzed). Comets were analyzed for % DNA in the tail and bars represent the average of all cells within a given sample population. Error bars represent one SD from the average. An * denotes a statistical significance of p<0.0001 between samples as measured by unpaired t-test. C) Each sample set represented by an average in (B) was further analyzed for distribution of individual cells in that sample set within four categories of % tail DNA. Those categories were <10% tail DNA (white bars), 11–25% tail DNA (light grey bars), 26–50% tail DNA (dark grey bars) or >50% tail DNA (black bars). The number of comets in each category was then converted to a fraction of 100% and plotted.

Two independent comet experiments were conducted on HFFs infected for 48 h and then irradiated with 50 J/m^2^ of UV and allowed to repair for 2, 6 or 24 h. It should be noted that this UVC dosage was non-lethal to the cells, with no cell loss in any of the experiments. Cell counts with and without UVC were essentially identical over the entire timecourse. The graph in Figure 2B shows the data from one of these experiments; data from the other was comparable. Comets were scored using VisComet software. The average % tail DNA for the population of cells scored in a given set is represented in each bar (error bars represent one SD). Four populations are represented in the graph and are shown in different shades of grey: mock infected cells plus or minus irradiation (M+UV and M alone, respectively) and virus-infected cells plus or minus irradiation (V+UV and V alone, respectively). A representative image of each group is shown in Figure 2A. As expected, M alone cells (white bars) produced very limited comet tails, with an average of less than 10% tail DNA. The M+UV cells (light grey bars) had significantly increased % tail DNA at early times, indicating their ability to begin NER incision events was intact. Twenty-four h of repair returned the M+UV cells' % tail DNA toward the M baseline. Surprisingly, the V alone samples (dark grey bars) had elevated % tail DNA throughout the timecourse. This seemingly perplexing result was investigated further (see below). The V+UV samples (black bars) had a substantially larger % tail DNA than the M+UV cells as early as 2 hp irradiation. In contrast to the M+UV samples, the % tail DNA in the V+UV samples did not decrease during the ensuing 24 h period. In fact, the % tail DNA remained high through 48 h of repair time (data not shown). Throughout the timecourse, the V+UV samples had statistically significant increases in % tail DNA over their M+UV counterparts (as measured by unpaired t-tests and indicated by asterisks in the graph). The persistence of comet tails in the V+UV samples was examined further in BrdU pulse/chase experiments below.

The distribution of % tail DNA within each sample type was plotted to distinguish whether changes were occurring over the timecourse (Figure 2C - four ranges of % tail DNA are represented in shades of grey). The distribution shown represents the experiment in Figure 2B. The distribution plot shows the increasing percentage of M+UV cells with less than 10% tail DNA over the timecourse of repair. In contrast virtually all cells in the V+UV samples have very high levels of tail DNA (greater than 50%) for the entire timecourse. As mentioned above, unexpectedly, the V alone samples increased in tail DNA percentage over the timecourse.

### Comet tails in V alone samples represented replicating viral DNA, not virus-induced damage

The source of comet tails in V alone samples was a conundrum. Electrophoretically induced migration of uncoiled/relaxed DNA is measured by the comet assay as the formation of a tail (as reviewed in [49]). The body of comet assay literature strongly suggests that the relaxation associated with open replication forks and Okazaki fragments connected with replicating genomes could appear as tail DNA in this assay ([50] and references within). Several studies have shown that HFFs infected in G_0_ with HCMV arrest at or near the G1/S transition, resulting in the replication of viral, but not cellular, DNA within these cells [51]–[54]. To determine whether the increase in % tail DNA in V alone samples was possibly attributable to the previously observed specific DSBs induced in a subset of cells by the incoming virus inoculum [12] or, more likely, the increase in viral DNA replication over time, the comet assays were repeated exactly as previously described in two parallel sets of samples (eight groups in total).

**Figure 3:**
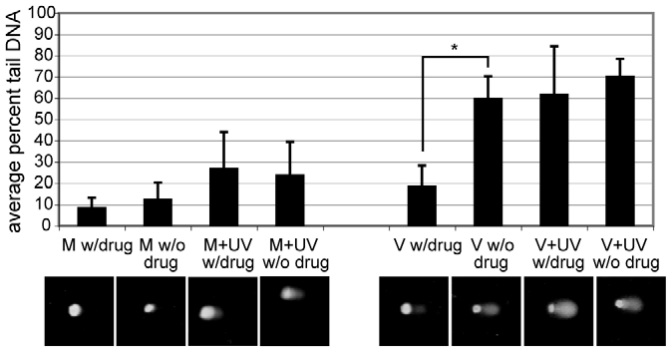
V alone tails were due to viral replication. Cells were treated as described in Figure 2 for comet analysis. Two parallel sets were prepared. One set received 45 µM ganciclovir continuously after 24 hpi. Cells were harvested at 24 h post irradiation for analysis. An * denotes a statistical significance of p<0.0001 between samples as measured by unpaired t-test. Below each category is an example of the comets observed for the given treatment.

Ganciclovir, a viral replication inhibitor, was added to one of the parallel sets of cells beginning at 24 hpi and throughout the remainder of the experiment. Addition of the drug at 24 hpi interrupted the infection at the pre-replication foci stage and any further development of these foci (and associated viral replication) in the treated samples was halted. All samples were irradiated at 48 hpi and harvested 24 h later. We reasoned that, if comet tails in the V alone samples were due primarily to viral replication, ganciclovir treatment should reduce comet tail levels toward M alone background levels. As can be seen in Figure 3, inhibition of viral DNA replication in the V alone samples dramatically reduced % tail DNA back toward M alone baseline levels. This reduction was the only statistically significant difference observed with the addition of ganciclovir to the samples (as measured by unpaired t-tests and indicated with an *). This result led us to conclude that the majority of the DNA in the V alone comet tails was not due to specific DSBs, but rather primarily due to viral replication.

### BrdU pulse/chase showed that high % tail DNA in V+UV samples was not due to viral replication

Interestingly, only nominal decreases in the % tail DNA were seen in the V+UV cells treated with ganciclovir. Our comet assay experiments with these cells were likely detecting the initiation of DNA repair in both host and viral DNA, not replicating viral genomes, however the ganciclovir experiments could not definitively distinguish if irradiation had inhibited viral replication during the repair cycle in the V+UV cells. Therefore, we assessed the extent of viral replication over time by BrdU-labeling the viral DNA before and at several points after UV-irradiation.

**Figure 4:**
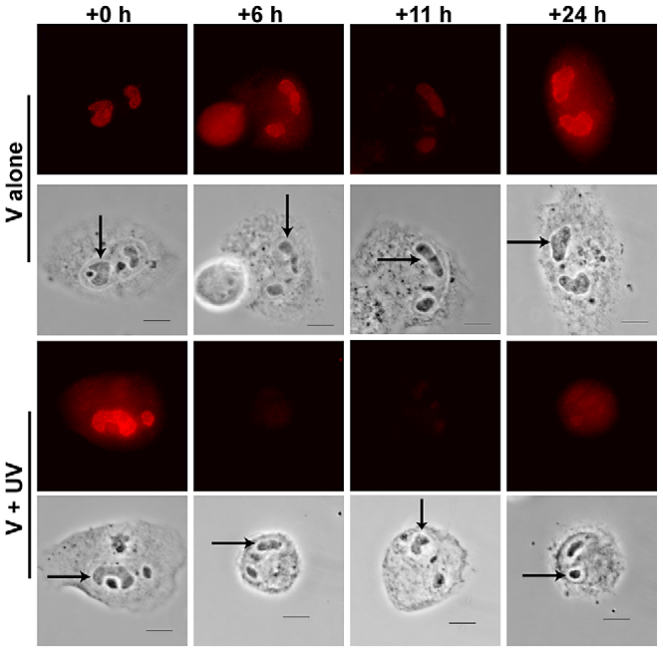
BrdU multipulse/chase experiments showed viral DNA replication was arrested after irradiation. Two plates of HFFs on glass coverslips were infected at an MOI of 5. One plate was irradiated at 75 J/m^2^ at 48 hpi and the other was left unirradiated Individual coverslips were pulse labeled with BrdU one h prior to harvesting (at −1,5,10 and 23 hpi, respectively). Coverslips were harvested at 0, 6, 11 and 24 hp irradiation. Cells were fixed and stained for BrdU incorporation (red) into viral RCs as described previously [68]. All images were captured using the same exposure time for direct comparison. Phase images of cells are pictured to show location of viral RCs within the nucleus (an example within each image is denoted by an arrow).

As described previously, cells were infected for 48 h on coverslips. Coverslips were then divided into two groups. One group was not irradiated and the second group received 75 J/m^2^ UV. In addition, one coverslip from each group was pulse-labeled with BrdU to provide a baseline of incorporation (and viral replication) (t = 0 h in Figure 4). The level of active viral replication in the two groups at each timepoint post irradiation was assessed by pulse labeling with BrdU (as described in Materials and Methods) just prior to harvesting coverslips (one each from the unirradiated and irradiated groups).

As seen in Figure 4, the unirradiated samples continued incorporating BrdU into the replicating viral DNA located in the RCs (upper panels, RCs are marked with arrows). However, after irradiation, viral replication essentially ceased in the irradiated samples (bottom panels, +6 and +11 h images show essentially no RC staining). A small amount of BrdU incorporation into the DNA in the RCs was seen in these V+UV cells after 24 h of recovery, but the amount of incorporation was nominal compared to their unirradiated counterparts. We also believe that the signal observable in the RCs at 24 hp irradiation in the V+UV samples is most probably due to BrdU incorporation into repair patches during unscheduled DNA synthesis (UDS) [55]. Small regions (of ∼20 nucleotides) must be resynthesized after removal of CPDs from the viral DNA. BrdU incorporation is commonly utilized in the DNA repair field to demonstrate repair has actually occurred in the nucleus of irradiated cells via UDS. These experiments demonstrated that viral replication was not responsible for the large % tail DNA in the V+UV comet assay samples.

### Residual UV dimers present after 24 h of recovery in V+UV samples were specifically localized in the cellular DNA

**Figure 5:**
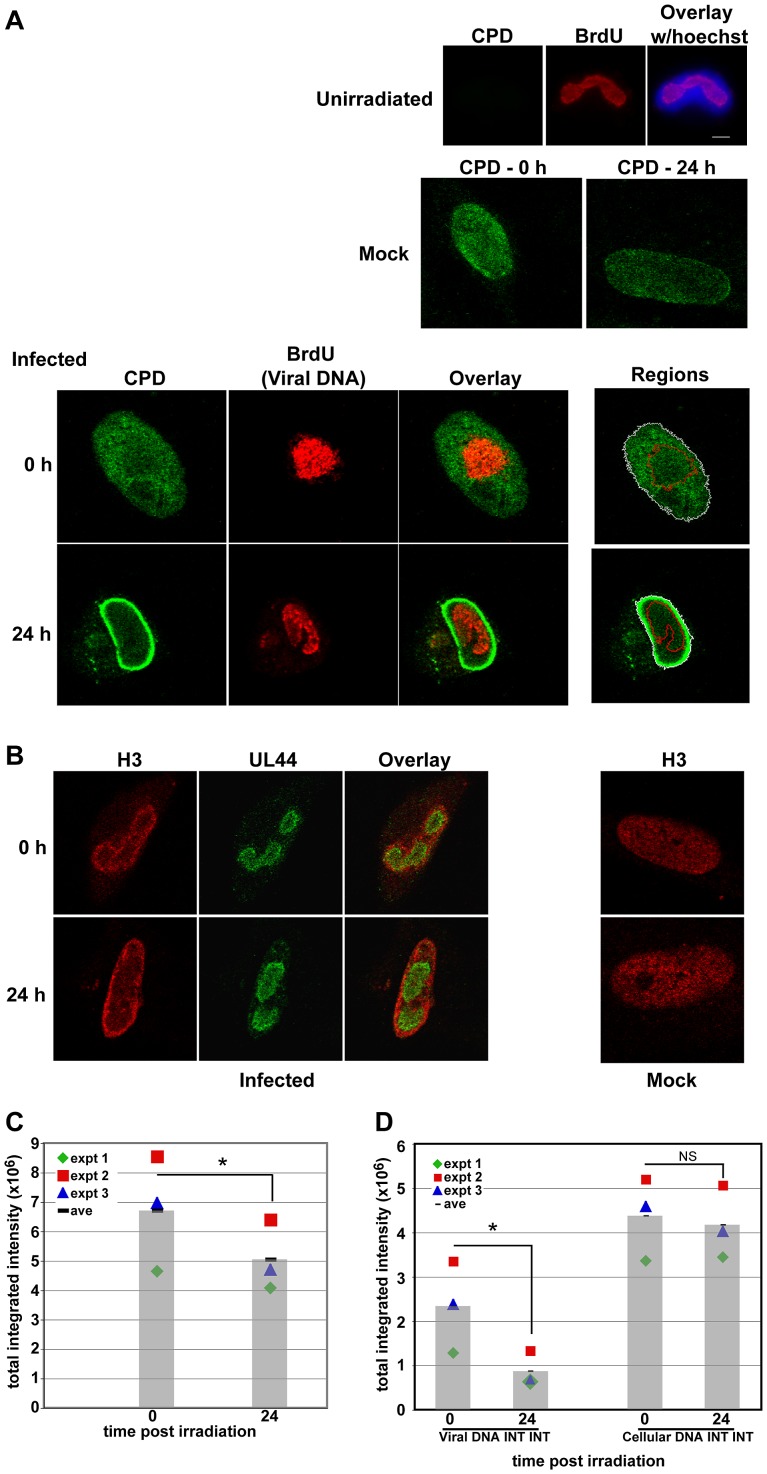
CPDs were selectively removed from viral DNA. Cells were seeded and infected as described in Figure 1. At 48 hpi, cells were irradiated (or unirradiated) with 75 J/m^2^ and harvested at the indicated times post irradiation. Cells were pulse-labeled with BrdU just prior to irradiation to enable viral RC visualization. After harvesting, cells were treated as described in Materials and Methods unless otherwise noted. A) Cells were stained for localization of CPDs (green) and DNA (BrdU - red) and imaged using confocal microscopy. Top panels show virus-infected cells with no irradiation to show the specificity of the CPD Ab. Middle panels show CPD staining of M+UV cells at 0 and 24 h post irradiation. Bottom panels show V+UV cells at 0 and 24 h post irradiation. Overlays show selective removal of CPDs from viral DNA over time. Far right panels illustrate how the regions were mapped for analysis in 5C and D. B) Parallel coverslips were harvested at 0 and 24 hp irradiation and fixed using standard “fix first” conditions. These coverslips were stained for visualization of cellular histone H3 (red) and viral processivity factor UL44 (green) to mark viral RCs. Left panels show V+UV samples, right panels show H3 staining in M+UV samples. Images were taken using confocal microscopy as in A. C) Quantitation of total nuclear INTINT at 0 and 24 hp irradiation for three separate experiments was performed as described in Materials and Methods. Average total nuclear INTINT for separate experiments are plotted as different symbols. Grey bars represent the average of these experimental points. The asterisk indicates the statistical significance (p-value<0.0001) between the CPD signal for the entire nucleus at 0 hp irradiation and at 24 h post irradiation (by mixed effects ANOVA). D) Further analysis of the components of the total INTINT (viral DNA and host DNA) by mixed effects ANOVA showed statistical significant differences in the viral DNA INTINT between 0 and 24 h (p<0.0002), but not the host INTINT (p>0.16). NS = not significant.

There appeared to be residual damage in the V+UV cells in comparison to M+UV cells. We therefore investigated whether there was specific localization of these dimers within the nuclei. An Ab developed by Dr. Toshio Mori [56] and specific for the most prevalent form of UV-induced damage, CPDs, was used to immunofluorescently visualize induced dimers *in situ* at 0 and 24 hp irradiation. Removal of CPDs at doses ranging from 30–75 J/m^2^ UV was examined. Confocal microscopy found both M+UV and V+UV nuclei (Figure 5A) stained for CPDs across the entire nucleus at time 0 h post irradiation. CPD adducts were formed in both cellular and viral DNA, with minor variations in intensity seen across an individual nucleus and from cell to cell. Cells were pulse-labeled with BrdU prior to irradiation to allow localization of viral DNA within the RCs for further quantitative analysis. Cells were stained with CPD- (green) and BrdU- (red) specific Abs simultaneously. M+UV and V+UV cells both had residual CPDs 24 hp irradiation. However, in the V+UV cells, dimers were localized specifically to the periphery of the nucleus and dimers were largely absent from the viral RCs (as localized by BrdU staining).

It has previously been shown that cellular DNA is marginalized to the edges of the nucleus at late times pi using histone localization [57]. We stained both M and V cells at 0 and 24 hp irradiation with an Ab to detect the localization of histone H3 and found, much like Monier and colleagues, that this cellular histone associated almost exclusively with DNA at the edge of the nucleus and outside of the RCs in infected cells (as marked by UL44), but across the entire nucleus in mock-infected samples (Figure 5B). This confirmed that residual CPDs were located primarily within the cellular DNA. CPDs appeared to be specifically removed from the viral RCs at all three doses of irradiation tested (Figure 5a shows only images of cells treated with 75 J/m^2^; identical images were obtained at lower doses, which are not shown). These results led us to believe that in permissively infected HFFs irradiated at 48 hpi, there was preferential removal of CPDs from the viral DNA.

The removal of CPD signal from these infected cells was quantitated over the 24 h period of repair. Images of infected cells dually-labeled for viral DNA (BrdU) and CPDs were captured at 0 and 24 hp irradiation using confocal microscopy. All images were captured using exposure times below which any pixels were saturated, including the brightest areas at the 24 hp irradiation V+UV cells' peripheries. Data from three separate experiments were analyzed using Metamorph software as described in the Materials and Methods. Briefly, after finding the center plane of each image, the RC area and the total area of the nucleus were defined and the integrated intensities (INTINT) of both regions were recorded. An example of the regions created by MetaMorph are shown mapped onto the infected cells in Figure 5A to illustrate the process. The RC region is outlined in red and the entire nucleus in white. Subtracting the INTINT of the RC from that of the entire nucleus determined the INTINT of the cellular DNA for each cell. For example, the intensity data for the cells shown in Figure 5A was: for the 0 h cell, Nucleus Integrated Intensity (NII)- 21.4 Million counts (M), Replication Center Integrated Intensity (RCII)- 5.4 M, Host Integrated Intensity (HII)- 16 M; for the 24 h cell, NII- 5.7 M, RCII- 1.2 M, HII- 4.5 M. Initial comparisons found differences in the total CPD INTINT signal within the nucleus at the two timepoints post irradiation (0 and 24 h). A mixed-effects ANOVA model was used to test these data for statistical significance. Using the different experimental dates as blocking factors to control for technical variation among the dates on which the experiments were performed, the results showed that the CPD signal for the entire nucleus was significantly greater at 0 h than at 24 hp irradiation (F = 20.7; df = 1, 121; p-value<0.0001). The averages for the three separate experiments are plotted (and represented by different symbols) in Figure 5C. The grey bars represent an average of the three separate experiments for ease of interpretation.

The statistically significant decrease observed in the total CPD signal prompted further analysis of the component viral and host DNA signals. Two post hoc tests were performed to determine if the decrease in the CPD signal found in the entire nucleus was independently attributable to CPD signal changes in either the host or viral DNA. The change in CPD signal in the host DNA and in the viral DNA were analyzed separately, again using a mixed effect ANOVA model to control for technical variation among dates while testing the effect of time on removal. After correcting for multiple statistical tests on the same data, the difference between 0 and 24 hp irradiation in the signal intensity within the host DNA was not significant (F = 3.1; df = 1, 123; p-value>0.16), whereas the difference between 0 and 24 hp irradiation in the signal intensity in the virus DNA was highly significant (F = 64.5; df = 1, 109; p-value<0.0002). Again, the averages for each experiment were plotted in Figure 5D, with the grey bar representing the average of the three experimental points. Although the individual experiments showed different average raw intensities, the downward trend for each experiment demonstrated a statistically significant removal of CPD signal from the viral DNA. These results clearly indicated that the decrease in the CPD signal in the nucleus following 24 h of repair was due to a decrease in the CPD signal in the virus DNA with no parallel decrease in signal within the host DNA.

### Selective removal of CPD signal from the RCs was not due to processing of viral DNA

**Figure 6:**
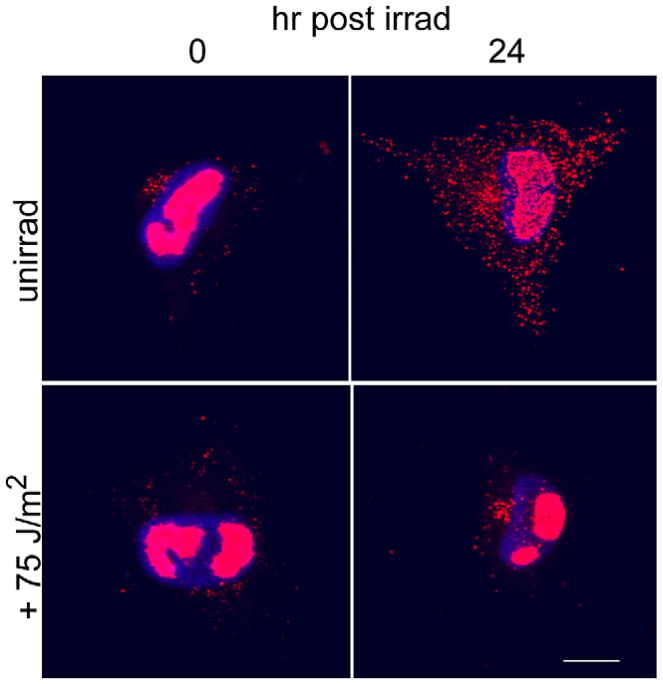
BrdU pulse and chase of viral DNA revealed no appreciable migration of pulsed viral DNA out of RCs during 24 h chase. HFFs were infected on coverslips. One h prior to irradiation at 48 hpi, infected cells were pulse-labeled with BrdU. One half of the coverslips were then irradiated with 75 J/m^2^. The second half was not irradiated. Timepoints were taken at 0 and 24 h post irradiation (or control treatment). Coverslips were fixed and stained for BrdU incorporation and imaged using confocal microscopy. Projection images of the entire stacks are shown, with Hoechst staining of the nuclei in blue.

Others have reported that viral DNA could potentially be replicated, packaged and transported out of the nucleus within a 24 h period [58]. To determine if specific removal of CPDs from the viral RCs was due solely to normal egress of the virus during active infection, the actively replicating virus within the RCs of HCMV infected cells was pulse-labeled with BrdU at 48 hpi. One half of the coverslips were irradiated with 75 J/m^2^ UV. It should be noted that experiments at 50 J/m^2^ produced identical results and are therefore not shown. Cells from both irradiated and unirradiated groups were harvested at 0 and 24 hp irradiation. All planes of a confocal image were projected into a single plane to gain a view of the entire cytoplasm and nucleus of an infected cell at the different times post BrdU pulse. Using these projected images, we could observe some movement of pulse-labeled virus-containing virions out into the cytoplasm of the unirradiated cells by 24 h post irradiation (visualized as individual spots of BrdU in Figure 6, top right panel). It should be noted that a significant fraction of the labeled viral genomes still remained within the RCs at this point. Conversely, we detected negligible movement of pulse-labeled viral DNA out of the RCs in the irradiated samples over the 24 h period (Figure 6, bottom right panel). This indicated that the decrease in CPD signal from the RCs observed in these cells was not caused by virus egress, but rather was due to selective removal of CPDs from the viral DNA.

### Dual-color visualization of virus and host DNA revealed selective repair of the viral genome

**Figure 7:**
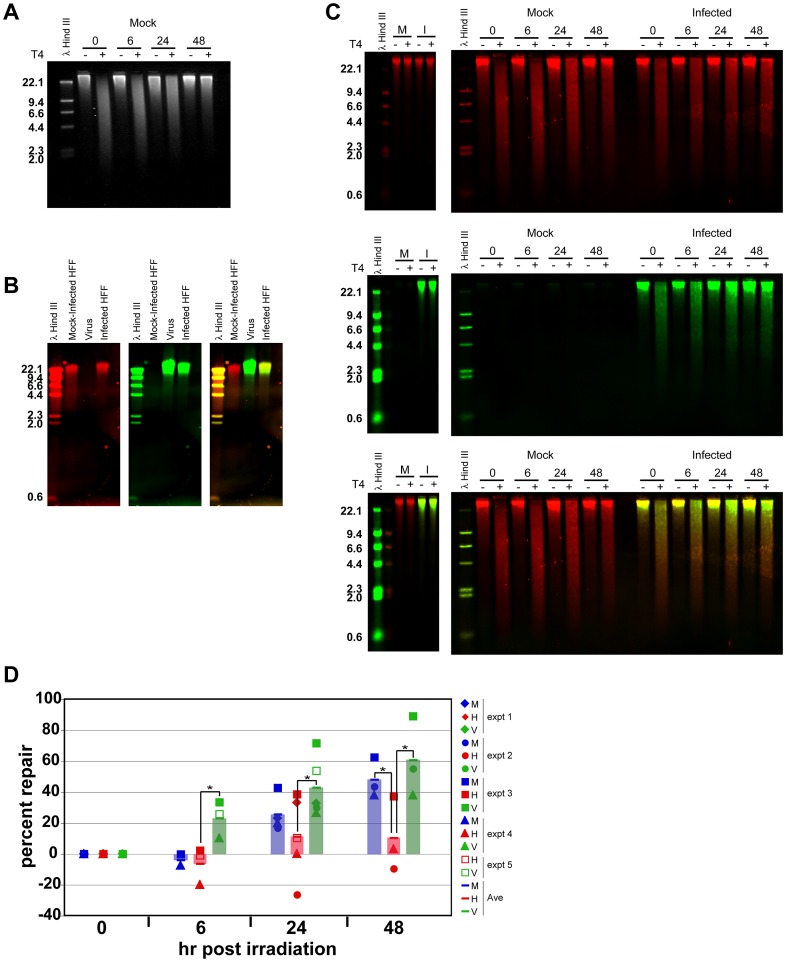
Dual color T4 assays revealed faster and more substantial repair of CPDs in viral DNA of infected cells. A) Mock-infected cells were irradiated with 50 J/m^2^ UV at 48 hpi and cells were collected at the indicated times post-irradiation for T4 assay analysis as described in Materials and Methods. Following T4 digestion, fragments were separated on an alkaline agarose gel, which was subsequently stained with SYBR Gold. Sizes of marker fragments are labeled in Kb in all images. B) DNA from mock-infected cells, pelleted virions and virus-infected cells were electrophoresed on a native agarose gel and transferred to nylon membrane. The blot was probed with digoxigenin –dUTP labeled host genomic probes (red - left panel) and biotin-dUTP labeled virus genomic probes (green- middle panel). An overlay image of red and green channels is shown at the right. Yellow indicates the presence of both viral and cellular DNA. Blots in B and C were visualized on a Li-Cor Odyssey scanner. C) HCMV-infected and mock-infected cells were irradiated (right panels) with 50 J/m^2^ UV or unirradiated (left panels) at 48 hpi. Cells were collected at the indicated times post-irradiation for T4 assay analysis as described in Materials and Methods. Following T4 digestion, fragments were separated on an alkaline agarose gel and transferred to nylon membrane. The blot was probed simultaneously with digoxigenin –dUTP labeled host genomic probes (red - top panel) and biotin-dUTP labeled virus genomic probes (green - middle panel). An overlay image of red and green channels is shown in the bottom panel. Unirradiated panels show only 48 hp irradiation samples. D) Percent repair in mock-infected (blue) and virus-infected cellular DNA (red) and viral DNA (green) was quantitated as described in Bespolov et al [59]. The results from five separate experiments are represented as different symbols on the graph. Bars represent the average of these experiments for ease of viewing. Asterisks represent statistically significant differences using paired t-tests for host versus viral DNA repair (p<0.001, p<0.01, p<0.01, for timepoints 6,24 and 48, respectively) and for host versus mock DNA repair (p<0.02 at 48 hp irradiation). M = mock DNA, H = host DNA, V = virus DNA.

Global genomic repair of CPDs can be estimated using T4 endonuclease V cleavage analysis [59]. T4 makes a highly specific single strand nick 5′ of UV-induced CPD adducts [60]. Samples are then separated via electrophoresis on an alkaline agarose gel. In these T4 gels, DNA that is either undigested or unirradiated is visible as a distinct high molecular weight (HMW) band at the top of the lane. In contrast, UV-irradiated DNA subsequently digested with T4 and electrophoresed yields a smear of lower molecular weight fragments down the lane, indicating nicking at CPD lesions. Over the course of repair, the smear returns to a HMW band indicative of repaired, full length DNA. Until now, visualization of the cleavage products has typically been via ^32^P labeled probes [59], [61], [62] or ethidium bromide [63], [64]. In Figure 7A, we show an example of mock-infected samples irradiated at 50 J/m^2^ and then digested with T4, run on an alkaline agarose gel and stained with SYBR Gold, a ss and dsDNA binding dye, to illustrate these gels. It can be clearly seen that the undigested samples remain as HMW bands and the digested samples run as a smear in the gel. As repair occurs the length of the smear decreases and the HMW band returns, indicating removal of CPDs and religation of the DNA.

When quantitating the extent of DNA damage, the average fragment length of DNA within the lane is inversely proportional to the number of CPD lesions present within the sample, i.e.- the smaller the average fragment length the more T4 cleavage sites, and therefore CPD lesions, present within the sample. These techniques have been used extensively to study genomic [59], [63], [64] and gene-specific [61], [62] CPD repair in a variety of organisms. However, determining repair of virus and host genomic DNA independently within HCMV-infected cells proved more challenging. A traditional approach would probe, strip and reprobe a single Southern blot for host and viral DNA; however stripping introduces the potential loss of signal. We developed a new method to visualize both virus and host genomic DNA simultaneously on a single blot using a Li-Cor Odyssey infrared imager.

To develop the dual-color Southern technique, we ran DNA isolated from mock-infected HFFs, pelleted viral particles and infected HFFs on a native gel before blotting to nitrocellulose and probing with digoxigenin-labeled host probes and biotin-labeled viral probes (Fig. 7B). Host DNA was visualized in the red channel (685 nm) and viral DNA was visualized in the green channel (785 nm). In the merged image, the DNA from the infected cells appeared yellow, since infected cells contained both host and viral DNA, while the uninfected cellular DNA was red and the purified viral DNA was green.

After validating this dual-color Southern technique's ability to distinguish viral from host DNA, it was used to probe experimental T4 blots (Fig. 7C). The far left panels of Figure 7C illustrate unirradiated DNA digested with T4 on these gels. The visible bands are equivalent to the HMW bands in the irradiated, undigested samples shown in Figure 7A. The right-hand panels in Figure 7C show the irradiated samples run on alkaline agarose gels. An analysis of the single channel blots and the overlay in these right-hand panels display several readily discernible features. First, after 48 h of repair, there was substantially more HMW DNA in the mock +T4 lane when compared to the infected +T4 lane in the red channel indicating decreased repair in the host DNA of infected cells (cellular DNA- top panel). Second, analysis of the viral DNA in the infected cells also showed a substantial return of a HMW band after 48 h of repair, indicating efficient repair of UV-induced DNA lesions (+T4 lane, green channel, middle panel). Lastly, analysis of the 48 h infected cell +T4 lane in the overlay blot clearly showed a gradient of colors, with the HMW band being predominantly green (viral DNA) and substantially more red signal (cellular DNA) within the smaller molecular weight fragments of the smear (bottom panel). It is important to realize that although the decrease in the DNA smear was subtle in the mock-infected and viral DNA lanes, the reappearance of a “full length” product/band at the top of the lanes as the timecourse progresses was more significant and indicated dimer removal and completed repair. This band reappears convincingly in the mock-infected and viral DNA lanes, but is nominal in the host DNA within the infected cells.

In Figure 7D, the results from five biological replicate experiments are plotted using different symbols, with the average of these experiments represented by bars for ease of comparison. The data is represented as “percent repair” of the dimers in this graph using the quantitation protocol described in Bespalov *et al* [59]. There is considerable variability in the results for these five T4 experiments. The variability is on par with that found in both the comet assays and the CPD removal experiments. Use of a Stratalinker for UV irradiation may have contributed to this variability, as the data shows that the initial induction of CPDs was not entirely consistent across experiments. However, rather than confound our results, we found highly statistical differences between groups as detailed below.

As depicted in Figure 7D, mock-infected HFFs repaired an average of ∼50% of CPD adducts by 48 hp irradiation (Figure 7D, blue bars). In contrast, host CPD repair in HCMV-infected cells plateaued at an average of ∼10% by 24 h and remained constant through 48 h of repair (red bars), while within the same cells, an average of ∼60% of CPDs were repaired from within the viral DNA (green bars). Statistical analyses were performed for each time point comparing the mean repair of the host DNA versus the viral DNA (or versus mock DNA) using one-tailed paired t-tests. A paired t-test controls for variation between experiments as well as unequal variances between the two measures (host versus viral or mock DNA). At each time point, the amount of viral DNA repair was statistically greater than the amount of host DNA repair (p<0.001, p<0.01, p<0.01, for timepoints 6, 24 and 48, respectively using one-tailed paired t tests). Statistically significant differences between the host and mock DNA repair were only observed at 48 hp irradiation (p<0.26, p<0.30, p<0.02, for timepoints 6, 24 and 48, respectively). These significant differences are indicated by asterisks in 7D. Therefore, repair of viral DNA was initiated more quickly and progressed more rapidly than repair of the host DNA within the same cells. The differential in repair of host and viral genomic DNA in infected cells confirmed our IF observation that CPDs were selectively removed from the viral DNA, but remained in the host genomic DNA.

## Discussion

The work reported here was based on the observed differential association of cellular repair proteins with viral RCs within the nucleus. We hypothesized that this association could favor viral repair and more importantly, be detrimental to repair of cellular DNA. To test this premise we UV-irradiated infected cells and then analyzed the removal of UV dimers by three methods; comet assays, IF localization of CPDs and dual-color T4 assays. Comet assays revealed that although infected cells were capable of mounting a repair response, they were unable to complete repair of all of the exogenously introduced damage. *In situ* localization of the CPDs showed that residual damage was confined to the cellular DNA. Lastly, dual-color T4 assays revealed faster and more significant repair of CPDs in the viral DNA than the host DNA within infected cells.

Over the past decade a great deal of work has focused on interactions of viruses and their host's DNA damage signaling molecules and repair machinery. Many of these studies (including our own [18]) have examined the triggering of ATM- and ATR-mediated DDRs by both DNA viruses and retroviruses (as reviewed in [16]). Certain viruses (for example, Adenovirus) actively thwart these damage responses, while other viruses (like HIV) require a DDR to replicate to full capacity. These studies have been informative and have discovered specific viral interactions with key players in these repair pathways; however they have not assessed the ramifications of infection upon the cell's subsequent ability to repair further insult to its DNA.

A number of studies have analyzed a cell's repair capabilities following infection. These studies include the repair of exogenously introduced damage in the cellular DNA in the context of single viral protein expression [21]–[32] and the effects of a complete infection [33]–[40], [65]. These papers have examined the capacity of the cell's homologous recombination, base excision, nucleotide excision and non-homologous endjoining repair machinery to function, with the very large majority of the investigations finding decreased capacity of the cell to repair damage after viral protein expression (or full infection) commenced. Only four of these studies have reported evidence of an increase in repair capacity of the cell after infection or viral protein overexpression [21], [23], [36], [40]. Our results extend this analysis and separate the two genomes within an infected cell. We demonstrate that, at least in the context of HCMV-infected fibroblasts, there is increased repair of UV-induced CPDs in the viral DNA, without a corresponding increase in repair of the host DNA.

In the next few paragraphs we will focus on the above studies most pertinent to our own results, emphasizing studies examining interactions with the NER machinery and/or with HCMV infection's influence on cellular damage repair. Several studies have utilized expression of single viral proteins in the analysis of UV damage. Expression of the Hepatitis B X protein (HBX) in different cell types [22], [25], [27], [29], [32] or expression of the Epstein Barr virus proteins EBNA3C or LMP1 in transfected cells [26] decreased repair efficiency of UV-induced damage in transfected cells.

More pertinent to our study was that of Liang and colleagues [28], which used a herpesviral protein (γ herpesvirus 68 protein M2) and methodology similar to our own. Mouse 3T3 cells expressing M2 were assessed for the ability to repair exogenously induced UV damage. At low dosage (2.5 J/m^2^) M2-expressing cells' capacity to remove dimers was decreased, which was most pronounced at 24 hp irradiation. More dramatically, at 30 min post irradiation at very high dose (5000 J/m^2^) M2-expressing cells formed no comet tails, indicating they did not even initiate repair. Using a dimer-specific Ab they saw dramatically reduced dimer removal in the M2-expressing cells. Liang's results indicate that an M2-expressing cell had impaired ability to repair exogenous damage in host DNA via NER. We wonder if viral DNA would have been preferentially repaired if it had been present in these experiments?

An additional three studies have looked at NER repair in the context of full infection. Duong and colleagues [35] found reduced efficiency of Hepatitis C-infected cells to reactivate (and therefore repair) transfected UV-irradiated reporter plasmids (compared to uninfected control cells). Similarly, Philpott and Buehring found that multiple HTLV- and bovine leukemia virus-transformed lines (as well as cells transformed with just the HTLV Tax protein) had a decreased ability to repair a reporter construct damaged by UV [39]. Bowman and colleagues [65] looked at the removal of CPDs from host DNA during SV40 infection using dimer-specific Abs in slot blot analysis and found a decreased removal of these adducts. As in our studies, they utilized T4 assays to examine removal of damage from both the transcribed (transcription-coupled NER) and the non-transcribed (global genomic NER) strands of a cellular gene, DHFR. Interestingly, they found that repair of only the non-transcribed strand of DHFR was affected by SV40 infection, indicating that repression of p53 by SV40 might be involved (discussed further below). Once again, the question remains whether analysis of the SV40 DNA would have revealed increased and more rapid repair of the viral DNA in these cells.

The last set of papers that should be addressed deal specifically with repair in HCMV-infected cells. The literature has revealed varying effects of damage, depending upon the system being examined. Ranneberg-Nilsen and colleagues examined the capability of HCMV-infected human embryonic lung fibroblasts (infected under conditions similar to our study) to carry out BER [40], and found approximately twofold changes in repair, with different substrates being removed with greater or lesser efficiency. Studies from our own lab [36], using the same fibroblasts and HCMV isolate (Towne) as used in the current study, found that homology directed repair (HDR) was more efficient after infection, regardless of whether the reporter construct was integrated into the host cell genome or expressed transiently. Thus, neither BER nor HDR was affected as significantly as we have found NER to be.

Two additional works address the effects of HCMV infection on the introduction and frequency of DNA chromosome anomalies induced by subsequent exposure to genotoxic agents. The first [33] infected non-permissive peripheral blood lymphocytes (PBLs) with HCMV at low MOI in the presence of camptothecin and observed a synergistic increase in chromosome damage (including chromosome breaks), even in the absence of viral gene expression. These findings support our supposition that the repair of multiple forms of damage is inhibited in HCMV-infected cells. A separate study by Deng and coworkers [34] used freshly stimulated PBLs infected with HCMV at a higher MOI of 4. Their findings suggested that HCMV infection sensitized the chromosomes to drug-induced damage. Deng and coworkers' observation indicated that chromosome anomalies were present even without *de novo* viral gene expression in the non-permissive PBLs. This result is consistent with our earlier findings [12] that *de novo* viral protein expression was not required to induce site-specific chromosome damage. Our earlier results also indicated that certain virion-associated proteins cannot only induce damage, but may also interact specifically with the damage machinery to inhibit its operation.

These last studies have suggested experiments we intend to pursue in the future. First, does the same decrease in repair of cellular DNA occur if there is no replication of viral DNA within cells? This could be determined in non-permissively or semi-permissively infected cells by ascertaining whether a set of viral proteins and/or viral RC association of cellular proteins needs to occur for this effect to be observed. The results of the ganciclovir experiments shown in Figure 3 suggest that establishment of fully functioning replication centers may not be required for negative effects on cellular NER repair. Second, would the same decreases in repair capacity be seen in latently infected cells or cells with limited viral replication (such as long-term infected neurons [66])? Third, does the presence or absence of the p53 protein play a role in repair of different types of damage within infected cells? Certainly the reports of others [29], [32], [65] mentioned above indicate that, at least in the context of repair of UV-induced damage, interactions of the virus with p53 might influence global genomic repair within the cellular DNA. Our earlier studies have shown clear interactions with, and the importance of, p53 to HCMV replication [67]–[69], indicating p53 may play a role in the selective repair of viral over cellular DNA.

Our study is not the first to look at the capacity of an infected cell to repair exogenously introduced DNA damage. However, utilizing novel techniques, our experiments assessed initiation of repair, removal of CPDs and repair of the DNA substrate in both the cellular and viral DNA separately. Comet assays indicated that infected cells were fully capable of initiating repair, but still retained residual damage 24 hp irradiation. Confocal images of infected cells with separately labeled viral DNA (using BrdU pulse-labeling) showed definitive removal of CPD signal from viral DNA in the RCs but no statistically significant removal from the host genome. Importantly, this indicated the residual comet tail damage observed in the V+UV samples was due to persistence of CPDs in the host DNA and not in the viral genome. Additionally, development of a dual-color Southern methodology has allowed utilization of the well-established T4 assay to analyze two separate DNA genomes simultaneously. These dual-color T4 assays demonstrated faster and more significant repair of CPDs from the viral DNA than the host cellular DNA within the same cell. It is our belief that the compromised capability of infected cells to repair damage may ultimately be manifested in the induction of CNS defects in the HCMV-infected neonate. Future studies will extend this avenue of investigation.

## Materials and Methods

### Cell culture conditions

Primary human foreskin fibroblasts (HFFs) (a gift from Steven Spector, UCSD) were isolated from tissue and propagated in Earle's minimal essential media (MEM) supplemented with 10% heat inactivated fetal bovine serum (FBS), L-glutamine (2 mM), penicillin (200 U/ml), streptomycin (200 µg/ml), and amphotericin B (1.5 µg/ml). Cells were grown in humidified incubators maintained at 37°C and 5% CO_2_.

### Viral infection conditions

G_0_ synchronized HFFs were trypsinized, counted, reseeded at a lower density and allowed to settle for approximately 2 h. Cells were infected at a multiplicity of infection (MOI) of 5 with the Towne strain of HCMV, obtained from ATCC (#VR 977). Two to four hpi, virus inoculum was removed and cells were refed with media and allowed to incubate as described below. The virus was propagated under standard procedures [70].

### Immunolocalization of nucleotide excision repair (NER) proteins

HFFs were mock- or virus-infected as described above. At 48 hpi, coverslips were harvested for colocalization of cellular NER proteins with the viral processivity factor, UL44. Coverslips were treated in one of two ways. In the first method, cells were extracted-first in a CSK buffer solution (10 mM Pipes, 100 mM NaCl, 300 mM sucrose, and 3 mM MgCl_2_) containing 0.5% Triton X-100 [41]. Cells were then rinsed in CSK twice and fixed with 3% formaldehyde in PBS (with 0.5 mM MgCl_2_, and 0.5 mM 3 mM CaCl_2_) for 10 min. In the alternate method, coverslips were extracted using standard formaldehyde fixation and Triton X-100 extraction as described previously [69]. See the Results section for further discussion of “fix first” versus “extract first” conditions and the information that can be gleaned from use of these different methods. Incubation of coverslips with Abs and mounting for examination were as described previously [69]. Nuclei were counterstained with Hoechst dye. The images of NER protein localization were obtained using a Nikon Eclipse E800 fluorescence microscope equipped with a Nikon DXM camera and Metavue software. Primary antibodies (Abs) used in Figure 1 and Table 1: mouse monoclonal Abs to XPB and XPD were kind gifts of Jean Marc Egly [71], [72]; mouse monoclonal Abs to XPA (2A4), XPG (8H7) and ERCC1 (3H11) and rabbit polyclonal Abs to XPC (RW028) and XPF (RA1) were kind gifts of Rick Wood [73]–[76]; mouse monoclonal Ab to UL44 (1202S - Rumbaugh Goodwin Institute); rabbit polyclonal Ab to CSB (Santa Cruz Biotechnology). Secondary Abs used in Figure 1A were donkey anti-rabbit TRITC-coupled Ab (Jackson Immunoresearch) and goat anti-mouse IgG1 alexafluor 488-coupled Ab (Molecular Probes). Secondary Ab used in Figure 1B was goat anti-mouse IgG FITC-coupled Ab (Jackson Immunoresearch).

### UV irradiation experiments and subsequent comet assays

HFFs were infected as described above. At 48 hpi, cells were washed in PBS and one set of mock and viral plates were irradiated in a Stratalinker 1800 at a dose of 50 J/m^2^. A second set of plates was left unirradiated. Irradiated cells were rinsed again, re-fed with media and allowed to recover for different periods of time (2, 6 and 24 hp irradiation). At the given timepoints, cells were washed once in cold PBS then scraped into cold PBS in microfuge tubes. Cell suspensions were adjusted to 1.5×10^5^ cells/ml. 50 µl of suspension was added to 500 µl of low melting point agarose (1% in PBS) and 75 µl of this suspension was placed in a thin layer on a coated glass slide (Trevigen). The agarose was allowed to gel at 4°C for 15 min. Cells were then lysed for 30 min *in situ* in a high salt/detergent solution (2.5 M NaCl, 1% sodium lauryl sarcosinate, 1% Triton X-100) at room temperature. DNA was denatured by treatment in alkali solution (pH>13) for 40 min. Prepared slides were placed in an electrophoresis tank filled with the above alkali solution. Very low current (280–290 mA) was applied to the tank for 20 min. Slides were dehydrated in EtOH, stained with Sybr Green (which binds to both ss and ds DNA) and visualized/photographed using a Nikon E800 Eclipse microscope equipped with a Nikon DXM camera and Act One software. VisComet software was used to analyze 50–100 cells/sample set (except where noted) of mock (M alone), viral (V alone), mock+UV (M+UV) and viral+UV (V+UV) at the given timepoints post irradiation. Comets were analyzed for % DNA in the tail. Data in Figures 2 and 3 are represented as the average of % tail DNA for the given sample set. Error bars represent one SD from that average. Each experiment was performed twice, with the data from a representative experiment shown in the figures. Unpaired t-tests were performed to assess the statistical significance between sample sets using GraphPad statistical software as noted. To distinguish whether changes were occurring over the timecourse, the distribution of % tail DNA within each sample type (M alone, V, alone, M+UV, V+UV) at the three different timepoints was plotted. In this plot, the percentage of DNA in the tail for each comet analyzed in a sample set was assessed and assigned to one of four categories (<10% tail DNA, 11–25% tail DNA, 26–50% tail DNA or >50% tail DNA). The number of comets in each category was converted to a fraction of 100% and plotted.

### Immunolocalization of dimers using UV Abs and BrdU labeling of viral DNA

Synchronized cells were reseeded into plates containing glass coverslips and infected as described above. At 48 hpi, cells were irradiated (or unirradiated) with 75 J/m^2^ UV and harvested at the indicated times post irradiation. Cells were also pulse-labeled with BrdU just prior to irradiation. BrdU labeling enabled viral RC visualization (as described previously [68]- 30 min pulse followed by 30 min chase in fresh media). After harvesting, cells were treated according to the methods in [77], which exposes both UV dimers and BrdU residues. Briefly, cells were fixed in ice cold MeOH: Acetic Acid (3∶1) for 20 min and subsequently washed in cold 100% EtOH. DNA was denatured for 3 min at room temperature (RT) using 70 mM NaOH dissolved in 70% EtOH. Finally, cells were washed extensively in PBS and stored at 4°C until staining.

Incubation of coverslips with Abs and mounting for examination were as described previously [69]. Cells were counterstained with Hoechst dye to visualize the nuclei. Mouse monoclonal Ab specific for CPDs has been described previously [56]. BrdU residues incorporated into viral DNA were stained with anti-BrdU rat monoclonal Ab (Harlan Sera-Lab). Cells stained for CPDs (detected with goat anti-mouse IgG2A Alexafluor 488 from Molecular Probes) and BrdU (detected with donkey anti-rat TRITC secondary Ab from Jackson Immunoresearch) were analyzed and photographed on an Olympus Fluoview 1000 confocal microscope using a 60× Plan Apo oil objective lens (1.42 NA). Care was taken to avoid the presence of saturated pixels within the images. Samples were excited using 405 nm (for BrdU), 488 nm (for CPD) and 561 nm (for Hoechst) laser lines. Images showing unirradiated samples stained for CPDs and BrdU were captured using the Nikon E800 Eclipse and Metavue software mentioned above.

In parallel, coverslips were harvested using “fix first” conditions as described above. These coverslips were stained with a polyclonal rabbit Ab to histone H3 (Millipore #06-755 detected using donkey anti-rabbit TRITC-coupled secondary Ab (Jackson Immunoresearch). They were also stained with the above-mentioned Ab to UL44 (detected using goat anti-mouse IgG1 AlexaFluor 488 (Molecular Probes)) to localize the RCs. These coverslips were blocked in 30% human IgG (instead of FBS) to inhibit non-specific binding of the rabbit Ab to the viral assembly complex within the cytoplasm of infected cells.

### Quantitation of changes in CPD intensity between 0 and 24 hp irradiation in confocal images

Image preparation and data generation were performed using MetaMorph (MM) Software (Universal Imaging). Stacked confocal images were captured as TIFF images on an Olympus Fluoview 1000 using 0.41 µm stepping. Twenty to thirty cells were analyzed per experiment, per time point as described below. Three separate experiments were analyzed. Using MM software, the center plane of each cell was identified from the stack of confocal images. The center plane was defined as the largest cross-sectional area of the virus RC. The image containing the center plane for each cell was color separated. The red (BrdU) and green (CPD) channels were saved as new images. This was performed for each individual cell, including all cells from images containing multiple cells. Thresholding of each color-separated image was used to define contiguous regions (in MM defined as Object(s)) for each nucleus and RC (many cells contained multiple RCs). Regions of Interest (ROIs) were created/saved surrounding these Objects (using the MM create ROI around Objects function). The CPD Integrated Intensity (INTINT) for each entire nucleus ROI was recorded. The ROI(s) of each RC(s) was mapped onto its corresponding CPD nucleus image. The associated CPD INTINT of each RC(s) region was recorded. A total RC CPD INTINT for cells containing multiples RCs was summed from that cell's multiple RC CPD INTINTs. The CPD total for the host cellular DNA was defined as all CPDs outside of the RC(s) (e.g. entire Nucleus CPD (-) Virus RC CPD = Host CPD). This data was analyzed using a mixed-effects ANOVA model (SAS, Cary, NC) comparing total CPD INTINTs between the 0 and 24 h post irradiation time points as described in the text.

### Drug treatment

Cells were treated as described above for comet analysis (irradiation of 50 J/m^2^ at 48 hpi). However, sample sets (M alone, M+UV, V alone, V+UV) were performed in duplicate. One of the sets continuously received 45 µM ganciclovir (after 24 hpi to inhibit viral DNA replication) and the second set a vehicle control. Cells were harvested at 24 h post irradiation for comet analysis as described above. For these experiments, 25–50 comets were scored per sample set. The experiment was repeated twice, and a representative sample set is shown in Figure 3.

### BrdU multipulse/chase experiments

Two plates of HFFs on glass coverslips were infected at an MOI of 5. After 48 h, one coverslip from each plate was removed and pulse-labeled with BrdU for 30 min and then chased for an additional 30 minutes in fresh media. These two coverslips served as time +0 h for the irradiated and unirradiated plates, respectively. After the chase period, one of the BrdU-labeled coverslips (and the remaining coverslips from its plate of origin) was irradiated at 75 J/m^2^. The other BrdU-labeled coverslip (and its partners) were left unirradiated. Time +0 h coverslips were then harvested. One h before each subsequent timepoint (at 5,10 and 23 hpi, respectively), an additional coverslip from each plate was removed and pulse-labeled with BrdU in preparation for harvesting at the appropriate timepoints (6, 11 and 24 hp irradiation). Cells were fixed and stained for BrdU incorporation into viral RCs as described previously [68]. Images were captured using the Nikon E800 Eclipse and Metavue software mentioned above.

### BrdU pulse and chase of viral DNA

HFFs were infected on coverslips as described above. After 47 hpi, one h prior to irradiation at 75 J/m^2^, infected cells were pulse-labeled with BrdU (and then chased for 30 min as described above) to label viral DNA within the RCs. Half of the coverslips were then irradiated with 75 J/m^2^; the second half was not irradiated. Timepoints were taken at 0 and 24 h post irradiation (or control treatment). Coverslips were fixed and processed for BrdU localization as described previously [68]. Cells were analyzed on the Olympus confocal microscope described above. Each Z-series was subsequently projected using the Olympus FSW software option of ‘Duplicate as displayed’ to create a single plane, 8-bit image for Figure 6.

### Preparation of probes for Southerns

Viral supernatants were centrifuged through a 25% sucrose (in PBS) cushion at 23, 000 rpm for 70 min at 10°C to pellet viral particles. Genomic DNA was extracted from HFF cells and viral particles as described previously [78]. *Hind*III-digested viral and HFF DNA were labeled with biotin-16-dUTP (Roche) and digoxigenin-11-dUTP (Roche), respectively, using the BioPrime Array CGH genomic labeling module (Invitrogen).

### Dual color Southern

The Li-Cor Odyssey Southern protocol was modified as follows. DNA was separated on a 1% native agarose gel. The DNA was depurinated for 15 minutes in 0.25 N HCl then denatured in 0.5 M NaOH and 1.5 M NaCl prior to transferring by capillary action onto 0.45 µm Magnacharge nylon membrane (GE water and process technologies) in 20× SSC (pH 7.0). After UV crosslinking, the membrane was prehybridized in a solution containing 5× SSPE, 2% SDS, 10% dextran sulfate, 1× Denhardt's solution and 10 µg/ml sheared, denatured salmon sperm DNA for 2–4 hours at 65°C. Labeled probes were boiled for 5 min and then rapidly chilled on ice for 10 min before addition to the prehybridization buffer and hybridization for 16 h at 65°C. The membrane was washed twice for 5 min in 2× SSPE at RT, twice for 15 min in 2× SSPE with 1% SDS at 60°C, and twice for 15 min in 0.1× SSPE at 60°C. The blot was blocked in 0.6% cold water fish skin gelatin (Sigma) in TBS with 0.5% Tween-20 (TBST) and 1% SDS for 1 h at RT. Anti-digoxigenin Ab (Sigma) was diluted 1∶1000 in 0.6% cold water fish skin gelatin in TBST and the blot was probed for 1 h at RT. The blot was washed at RT for 5 min in TBST, 10 min in TBST with 1% SDS, and three times with TBST for 5 min. Anti-mouse IRdye700 and streptavidin IRdye800 (Rockland) were diluted 1∶4,000 and 1∶20,000, respectively in 0.6% cold water fish skin gelatin in TBST with 0.02% SDS and the blot was incubated 45 min in the dark at RT. The blot was washed at RT for 5 min in TBST, 15 min in TBST with 1% SDS, three times with TBST for 5 min, and twice in TBS for 5 min. The blots were scanned using a Li-Cor Odyssey infrared imager (Li-Cor Bioscience).

### T4 Southern

HFFs were infected and irradiated at 50 J/m^2^ as described above. Cells were harvested at 0, 6, 24, and 48 h post irradiation. DNA was extracted as described above. 150 ng of DNA was digested with T4- or mock-digested and the digestions were loaded on a 1% alkaline agarose gel and separated at 25 V for 18 h as described previously [59]. The gel was neutralized for 45 minutes in 0.5 M Tris HCl pH 7.5 and 1.5 M NaCl prior to depurination, denaturation and capillary transfer as described above. Analysis of T4 Southerns for CPD removal was performed as described previously [59]. SYBR Gold stained gels were performed in the same fashion with the following exceptions: one microgram of DNA was loaded in each lane and gels were stained with SYBR Gold after neutralization. For statistical analysis, one-tailed paired t-tests were performed for each time point comparing the mean repair of the host DNA versus the viral DNA (or versus mock DNA) as described in the text.
